# Optimized Chitosan/Anion Polyelectrolyte Complex Based Inserts for Vaginal Delivery of Fluconazole: In Vitro/In Vivo Evaluation

**DOI:** 10.3390/pharmaceutics10040227

**Published:** 2018-11-12

**Authors:** Bayan Darwesh, Hibah M. Aldawsari, Shaimaa M. Badr-Eldin

**Affiliations:** 1 Department of Pharmaceutics, Faculty of Pharmacy, King Abdulaziz University, Jeddah 21589, Saudi Arabia; bdarwesh@kau.edu.sa (B.D.); haldosari@kau.edu.sa (H.M.A.); 2 Department of Pharmaceutics and Industrial Pharmacy, Faculty of Pharmacy, Cairo University, Cairo 11562, Egypt

**Keywords:** fluconazole, chitosan, alginate, lyophilization, polyelectrolyte complex, vaginal, microbiology, histology

## Abstract

(1) Background: Fluconazole, used orally for vaginal candidiasis, has reported gastrointestinal side effects. Therefore, researchers directed towards the drug vaginal delivery. However, vaginal delivery is limited by poor retention and leakage. Thus, this work aimed at exploring chitosan/anion polyelectrolyte complex (PEC) for the formulation of fluconazole vaginal inserts with controlled release and appreciable mucoadhesion. (2) Methods: PECs were prepared and assessed for interactions. Fluconazole PEC based vaginal inserts were prepared by lyophilization using mannitol. 3^1^5^1^ factorial design was applied to investigate the effect of the anion type and Chitosan/anion ratio on the inserts mucoadhesion and release properties. The optimized insert [based on 5:5 chitosan: anionic polymer (sodium alginate)] release was modulated by the release retardant; Compritol^®^ 888. The selected formulation was subjected to microbiological and histological evaluation. (3) Results: Fluconazole inserts showed satisfactory drug content, acceptable friability percentages and highest swelling indices at six hours. Statistical analysis showed significant effect of the studied factors on detachment force and release properties. Microbiological assays revealed significantly higher antifungal activity of inserts compared to fluconazole solution. Reduced inflammatory cells were confirmed by histological evaluation. (4) Conclusion: CH/Alg based vaginal insert could be a promising platform for vaginal delivery of antifungal drugs used for vaginal candidiasis treatment.

## 1. Introduction

Vulvovaginal candidiasis is a common fungal infection, where about 75% of healthy women suffer from at least one infection episode during their reproductive life span [1]. In healthy situation, lactobacilli dominate the vaginal flora maintaining an acidic pH through organic acids production. Any disturbance in the vaginal pH or disruption of the vaginal flora could potentially permit pathogenic microorganisms to grow [2]. *Candida albicans* is reported to cause over 80% of the diagnosed vaginal infections. Azole antifungals are considered as the most frequently used class for the treatment of vaginal candidiasis [3]. Fluconazole is a triazole antifungal that can be used for treatment of several fungal infections including vaginal candidiasis. Oral fluconazole administration is reported to cause many gastrointestinal side effects such as nausea, vomiting, abdominal pain and diarrhea, especially in case of prolonged duration of treatment [4,5,6]. The aforementioned limitations directed the researches’ attention towards the investigation of the vaginal route for the local delivery of the drug [7]. However, the administration via this route is generally associated with leakage of the formulation and low residence time at the vaginal cavity [8]. Recently, mucoadhesive systems showed potential in prolonging the residence time of ocular, nasal, vaginal and buccal drug delivery systems and consequently, sustaining drug release.

Chitosan (CH) is a linear cationic polysaccharide that is formed of *N*-glucosamine and small quantity of *N*-acetyl glucosamine [9]. It is obtained by deacetylation of chitin, a major component of sea crustaceans’ exoskeleton. Recently, it has gained attraction in drug delivery due to its mucoadhesive, permeation enhancing and controlled release properties. Moreover it is recognized as safe, biocompatible and biodegradable polymer [10]. Cationic nature of chitosan promotes interaction with anionic polysaccharide or synthetic polymer such as Na alginate (ALg), xanthan gum (XG) and carbopol (Carp) polymer [11].

Polyelectrolyte complexes (PECs) form between unlike charged polymer by the electrostatic interactions without the interference of chemical cross-linking agents. They allow drug entrapment resulting in altering the drug release and increasing its stability [12]. The electrostatic attractions between ionized amino groups of chitosan (NH^3+^) and carboxylic groups (COO-) of anionic polymer are the main interactions in the formation of the CH/anion complexes. For the past years, many researchers have studied CH/anion PEC as promising delivery systems for controlling drugs release [13]. Thus this work aimed at exploring the potentiality of CH/anion polyelectrolyte complexes for the development of fluconazole lyophilized vaginal inserts with controlled drug release, improved vaginal retention and consequently, enhanced antifungal activity and better patient compliance.

## 2. Materials and Methods

### 2.1. Materials

Fluconazole was kindly gifted by Spimaco, Riyadh, Saudi Arabia. Low molecular weight chitosan was purchased from Chemsavers, Bluefield, VA, USA. Na Alginate and xanthan gum were purchased from Sigma-Aldrich Chemical Co., St. Louis, MO, USA. Carbopol 971 was purchased from Lubrizol Co., Wickliffe, OH, USA. Compritol^®^ 888 ATO was purchased from Gattefosse, Lyon, France. All other reagents and chemicals used were of analytical grade.

### 2.2. Preparation of Fluconazole Polyelectrolyte Complexes (PECs)

Initially, solution of chitosan (1.5% *w*/*v*) was prepared in acetate buffer at pH 5, while solutions of anionic polymers (1.5 % *w*/*v*) were prepared in distilled water. Various volumes of anionic polymer solution were mixed with specified volume of chitosan to obtain different CH/anion ratios in the mixture ranging from 1:9 to 9:1 *w*/*w*. In each anionic polymer solution, fluconazole was dissolved in appropriate quantities to achieve a 20% (*w*/*w*) drug concentration in the final polyelectrolyte complex. The obtained mixture was then stirred using a mechanical stirrer at 1000 rpm for 24 h at room temperature. An accurately weighed amount of 2 g of the prepared mixture was poured into plastic tube and frozen at −80 °C for 24 h. The samples were freeze-dried (Lyophilizer Alpha 1-2 LD plus, Christ, Osterode am Harz, Germany) and the complexes obtained were stored in desiccators until use [14].

### 2.3. Differential Scanning Calorimetry (DSC)

Thermal properties of fluconazole, chitosan, anionic polymers, their physical mixture and PECs were evaluated using DSC-50 (Shimadzu, Koyoto, Japan). Using purified indium (99.9%), samples (around 5 mg) were sealed in a 50 mL flat-bottomed aluminum pans at a constant heating rate of 10 °C/min and analyzed at a temperature ranging from 30–300 °C.

### 2.4. Fourier-Transform Infrared Spectroscopy (FTIR)

FTIR spectra of pure fluconazole, chitosan, anionic polymers, physical mixture and PECs were recorded using signal/noise ratio (S/N) of 30,000:1. Potassium bromide (KBr) disc technique via FTIR spectrophotometer (Thermo Fisher Scientific, Waltham, MA, USA) was used for scanning spectral region of 4000 cm^−1^ to 400 cm^−1^ [15].

### 2.5. Scanning Electron Microscopy (SEM)

Morphological characteristics of fluconazole, chitosan, anionic polymers, physical mixture and PECs were analyzed using SEM (Quanta 450 FEG, Thermo Fisher Scientific). PEC was cut using a razorblade exposing their internal structure. They were then fixed and coated with gold–palladium under argon atmosphere [16].

### 2.6. Preparation of Vaginal Inserts

Lyophilized PECs containing 20% by weight fluconazole were used for preparing vaginal inserts. Mannitol was added for its bulking properties to enhance the strength of vaginal inserts during handling. Phosphate buffer solution, pH 4.5 was added to a specified weight of various complex/mannitol mixtures (9:1; *w*/*w*). This resulted in a wet complex, which was lyophilized in the form of cone-shaped inserts. The prepared inserts had an average diameter of 0.5 cm, height 2 cm and fluconazole content of 40 mg. The average weight of the final inserts were 221 ± 8.67 mg. Inserts were kept in a desiccator until further studies [16].

### 2.7. 3^1^5^1^ Factorial Design for Formulation and Optimization of PEC Based Vaginal Inserts

The influence of formulation parameters was studied using 3^1^5^1^ full factorial design, Table 1. The studied variables were the anionic polymer type (X_1_) used at three levels (Na alginate, carbopol, xanthan gum) and chitosan: anion ratio (X_2_) used at five levels (1:9, 3:7, 5:5, 7:3, 9:1) [16]. The formulations composition is shown in Table 1.The main effects of X_1_ and X_2_ on response variables including detachment force (Y_1_), release efficiency after 6 h of time (RE_6h_, Y_2_) and time after which drug release reached 63.2% (T_d_, Y_3_) were statically analyzed using Design-Expert^®^ software (version 11; Stat-Ease, Inc., Minneapolis, MN, USA, 2018). Analysis of variance (ANOVA) was applied to determine the significance of the studied factors. Numerical optimization following desirability approach was utilized to predict the composition of the optimized insert with minimized RE_6h_, maximized detachment force and T_d_.

### 2.8. Drug Content

Individual vaginal inserts were dissolved into phosphate buffer (pH 4.5). Continuous stirring of the solution was performed for 48 h, at 25 °C. Appropriate aliquots were filtered via a cellulose acetate membrane filter (diameter = 0.45 µm). Fluconazole concentrations were determined spectrophotometrically (UV spectrophotometer, Thermo Fisher Scientific) at the predetermined λ_max_ 260 nm [17]; each experiment was conducted five times. The Drug content was calculated using the following equation [18]:

% Drug content = (Actual amount)/(Theoretical amount) × 100(1)

### 2.9. Friability Studies

Friability study was performed to evaluate the inserts tendency to crumble or chip. Twenty vaginal inserts were sampled from each formula and exposed to 100 revolutions over a period of 4 min using a friability tester (Electrolab, Mumbai, India). Friability was computed as the percentage of weight loss of the insert after undergoing the revolutions. The friability of the prepared insert formulation were compared to the corresponding PECs [18].

### 2.10. Swelling Studies

Vaginal inserts water uptake was studied by weighing inserts and recording their weights (W1) before placing them separately in beakers. Specified volume of phosphate buffer (pH 4.5) was placed in each beaker and then placed in an incubator at 37 ± 0.5 °C. At specified time intervals, excess water was carefully removed using filter paper (0.22 µ) and the inserts were reweighed (W2). The experiment was repeated 5 times for each insert and the average W1 and W2 were recorded. Swelling index (SI) was calculated using the following equation [19]:

SI (medium uptake %) = (W2 − W1)/W1 × 100(2)

### 2.11. Ex Vivo Mucoadhesion Studies

The prepared inserts were applied on fresh cut sheep vaginal mucosa from slaughterhouse. The vaginal tissues cut in 1 cm^2^ were used within 12 h storage in refrigerator. The tissue was equilibrated at 37 °C for 15 min in phosphate buffer solution (4.5 pH) prior to the mucoadhesion test. The study was performed using Tensile Tester Machine (Shimadzu Co., Koyoto, Japan) [20]. The tissue was fixed on flat plate fitted to the lower grip of the instrument using glue. One cm^2^ of each insert was fixed to upper blade of the instrument and then kept contacting the mucosa for 5 min. After that, the detachment force needed to separate the insert from the mucosal tissue surface was recorded. Each experiment was repeated three times [21].

### 2.12. In Vitro Release and Kinetic Analysis of the Release Data

Fluconazole release was performed in 200 mL Phosphate buffer at pH 4.5 using USP Dissolution apparatus DT 720 Series (Erweka GmbH, Heusenstamm, Germany) at a temperature of around 37 ± 5 °C and 50 rpm speed. Aliquots were withdrawn at predetermined time intervals for a period of six hour. The amount of fluconazole was assayed spectrophotometrically at the predetermined λ_max_ 260 nm. Release studies were performed in triplicates [19]. Release efficiency after 6 h (RE_6h_) [14] and time to release 63.2% (T_d_) were computed for comparison between different formulations [22] using KinetDS 3.0 software (Free license software Rev. 2010, sourceforge.net). To study drug release kinetics from the prepared inserts, the release data was fitted into different mathematical models namely; zero order, first order, Higuchi, Hixson Crowell and Weibull model [23].

### 2.13. Release Profile Modulation

To further extend fluconazole release and decrease initial amount of drug released from the optimized insert (F3, 5:5 CH/Alg PEC), Compritol 888 was added in different percentages (0.5, 1 and 1.5 *w*/*w* %) as hydrophobicity imparting and release retarding additive yielding 3 insert formulations; F16, F17 and F18, respectively [14]. The vaginal insert that showed the desired release characteristics, F17, was selected for further investigation.

### 2.14. In Vitro Microbiological Evaluation

Evaluation of the antimicrobial activity of fluconazole vaginal inserts against *C. albicans* was performed. Colonies of *C*. *albicans*, grown under aerobic conditions in a medium of synthetic dextrose at 30 °C for 48 h, were used as controls [24]. Test was performed in a triplicate manner, using agar cup-plate method [25]. The cups (4 mm in diameter) were filled with one of the following preparations: the selected fluconazole vaginal, F17 [Fluconazole loaded CH/Alg complex (5:5, *w*/*w*) based vaginal insert with 1% Compritol], fluconazole solution (control 1), or unloaded vaginal insert based on the same composition of F17 (control 2). Subsequently, the inhibition zone of each cup was observed and mean inhibition zone radius was calculated for each preparation and compared among the different groups [26,27].

### 2.15. In Vivo Microbiological and Histological Testing

An animal model of experimentally induced vaginitis was used to study in vivo efficacy of fluconazole vaginal inserts. Female Wister albino rats (weight 200–230 g) were put in pseudo-estrus state, during which instrumental vaginal inoculation of *C. albicans* was done in order to induce a persistent infection. The experiment was performed according to international ethical guidelines for animal studies and was approved by the Research Ethics Committee of King Abdualaziz University (Reference No 502-17). Pseudo-estrus state was obtained by subcutaneous injection of medroxyprogestrone acetate (25 mg/rat) every 48 h during 6 days, then weekly [27]. Prior to experiment, cultures of vaginal fluid were performed to check the absence of Candida in all rats. Afterwards, groups of 6 rats were formed and their vagina were inoculated with 10^7^ blastoconidia/mL in 20 mL of sterile saline solution. To confirm infection, vaginal fluid was sampled after 2 days from the inoculation using sterile swabs and the number of colony units was counted to confirm the viability of the inoculum. On the same day (day 0), Infected rats were randomly allocated into one of the four following groups: Group 1 (G1) no treatment (control); Group 2 (G2) treated with one fluconazole PEC based vaginal insert, F17; Group 3 (G3) treated with one unloaded insert; and Group 4 (G4) treated with fluconazole solution. To assess treatment effect, vaginal swabs were collected and streaked over a Sabouraud dextrose agar plate which underwent 72-h incubation at 35 °C. Swabs were taken on the 2nd, 5th, 7th and 21st days following administration of the aforementioned treatments [24].

At the end of the 21st day, rats (one from each group and healthy rat) were sacrificed and their vaginal tissues were excised. The samples were fixed in 10% neutral formalin and then paraffin blocks and tissue sections were prepared. The specimens were stained with hematoxylin-eosin for histopathological assessment of the inflammation [28].

## 3. Results and Discussion

### 3.1. Differential Scanning Calorimetry (DSC)

**Figure 1:**
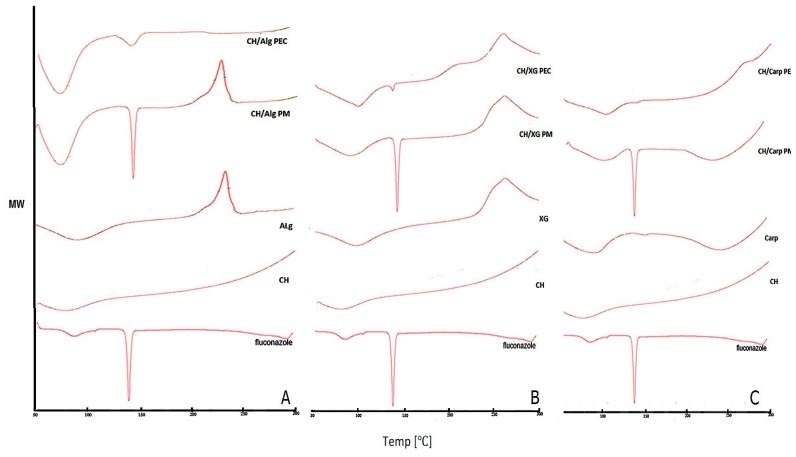
Differential scanning calorimetry (DSC) thermograms of fluconazole, chitosan, anion, their physical mixture (PM) and fluconazole CH/anion polyelectrolyte complex (PEC) (**A**) Na alginate (Alg) (**B**) Carpobol (Carp) (**C**) Xanthan gum (XG).

DSC was performed for pure fluconazole, polymers, their physical mixture and PECs to assess the physical interaction between the drug and different polymers used as shown in Figure 1. Fluconazole showed two peaks; broad peak at 76 °C and sharp intense peak at 139 °C corresponding to crystal dehydration and crystal melting points, respectively. Chitosan showed a broad endothermic peak at about 79 °C. Na alginate exhibited endothermal peak at around 80 °C followed by an exothermic peak at 240 °C, Figure 1A. Xanthan gum has an endothermal peak at 108 °C and an exothermal peak at 268 °C, Figure 1B, while carbopol had two broad endothermal peaks at 60 °C and 268 °C, Figure 1C. All the previous findings were in accordance with literature [29]. These distinctive peaks were preserved in all CH/anion physical mixtures suggesting the absence of interaction between polymers in their physical mixtures. Regarding the CH/Alg PECs, DSC showed disappearance of Na alginate exothermic peak at 240 °C; in addition to broadening and decreased intensity of fluconazole sharp endothermal peak, Figure 1A. However, in CH/Carp PEC, carbopol broad endothermal peak at 268 °C disappeared; as well as fluconazole sharp peak, Figure 1C. The aforementioned changes could probably indicate changes in some physical properties during complexes preparation such as the crystalline, dehydration or melting point. Disappearance of fluconazole peak might indicate the amorphization of the drug and/or its inclusion in the formed complex [14]. In case of CH/XG PEC, no change occurred to xanthan gum exothermal peak at 268 °C; moreover, fluconazole peak was preserved without broadening or shifting indicating absence or weak interaction. The observed decrease in fluconazole peak intensity could be due to dilution on mixing [14,16]. These results indicate strong interaction between Chitosan and both carbopol and alginate with possible complex formation and fluconazole inclusion, while absence or weak interaction in case of xanthan gum. 

### 3.2. Fourier Transform Infrared Spectroscopy (FTIR)

**Figure 2:**
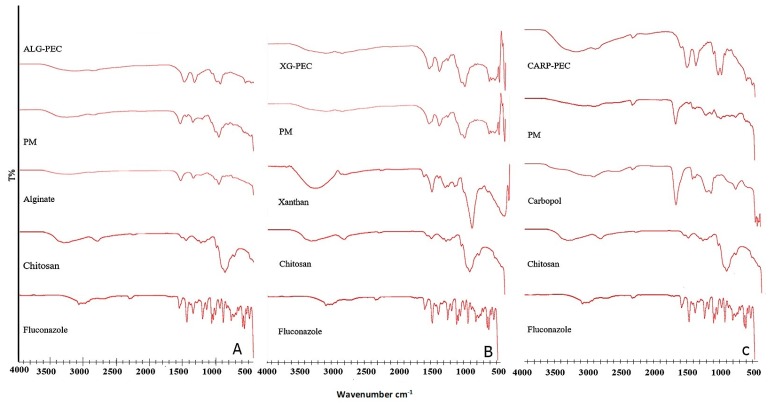
Fourier Transform Infrared (FTIR) spectra of fluconazole, chitosan, anion, their physical mixture (PM) and fluconazole CH/anion PEC (**A**) Na alginate (Alg) (**B**) Carpobol (Carp) (**C**) Xanthan gum (XG).

Examination of fluconazole spectrum reveals the presence of the characteristic bands of OH stretching in 3170, 1300 cm^−1^, as well as characteristic bands of C=C stretching in 1560 and 1620 cm^−1^ [15]. Chitosan showed the characteristic C=O band of amide at 1648 cm^−1^ and N=H band of amine at 1584 cm^−1^; in addition to characteristic N–H, C–N and N–H stretching bands for amine groups between 3300–3500 cm^−1^ [30]. FTIR spectra of Alginate Figure 2A, xanthan gum Figure 2B and carbopol Figure 2C showed typical C=O band of carboxylate at 1620–1700 cm^−1^ [14,30]. The IR spectra of the physical mixtures of chitosan with each of the anionic polymers appeared as a combination of the respective separate polymer spectra. Further, the physical mixture showed suppression of intensity of fluconazole peaks that could probably be attributed to Fluconazole dilution. The FT-IR of CH/Alg PEC showed disappearance of Alginate N–H peak along with a shift to the right of C=O peak from 1600 cm^−1^ to 1550 cm^−1^ Figure 2A, indicating strong interaction between chitosan and Na alginate by ionic bonds. In addition, FT-IR profiles of carbopol based PECs exhibited slight right shift of C=O band Figure 2C. This could be due to interaction between chitosan and carbopol. The changes observed in the characteristics fluconazole bands in both CH/anion spectra could highlight physical or weak ionic interaction between the complex and the drug molecule. On the other hand, CH/XG complex spectrum did not show any significant changes indicating weak interaction between polymers [14].

### 3.3. Scanning Electron Microscopy (SEM) Study

**Figure 3:**
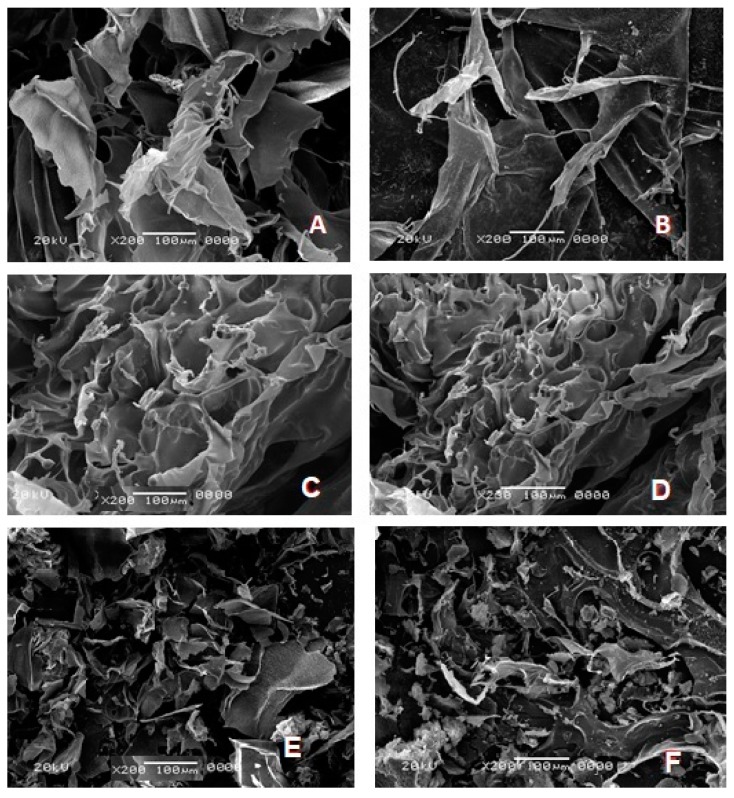
Scanning electron micrographs of (**A**) Unloaded 5:5 CH/Alg PEC—based insert (**B**) Fluconazole 5:5 CH/Alg PEC (**C**) Unloaded 5:5 CH/XG PEC—based insert (**D**) Fluconazole 5:5 CH/XG PEC (**E**) Unloaded 5:5 CH/Carb PEC—based insert (**F**) Fluconazole 5:5 CH/Carp PEC (×200).

Microscopic examination showed differences between unloaded and loaded PECs structural, Figure 3. Fluconazole-loaded PECs generally exhibited more porosity, surface roughness and sponginess compared to unloaded ones regardless of anion type [16]. By comparing anion types, it was observed that CH/Alg PEC and CH/Carp PECs matrix had more roughness and porosity with pores being of greater size than CH/XG, which has sponge like structure [30,31].

### 3.4. Characterization of Chitosan/Anion Complex Based Inserts

All the prepared vaginal inserts exhibited acceptable drug content, ranging from 95 ± 0.029% to 102 ± 0.057%. These results are in accordance with the pharmacopeial limit of (85–115%) [14,32]. Friability values for all inserts ranged between 1.91 ± 1.49% and 6.34 ± 2.91%, Table 1, which is in accordance with the acceptable range for lyophilized vaginal inserts previously reported in the literature [16]. The friability values were significantly lower than that of the corresponding PECs (ranging from 9.19 ± 2.03 to 23.12 ± 5.34, data not shown). These results emphasized the role of mannitol as bulking agent, which enhances mechanical strength of inserts upon handling [33].

The highest medium uptake ability for all the prepared vaginal inserts was observed at six hours ranging from 191.24 ± 1.07 to 215.98 ± 2.12%, 192.34 ± 2.42 to 209.61 ± 1.34% and 201.83 ± 1.78% to 221.75 ± 2.05% for CH/Alg, CH/XG and CH/Carp based inserts, respectively as shown in Table 1. One-way ANOVA showed significant difference among the three anions regarding medium uptake (*p* < 0.001). Post-hoc analysis showed that CH/XG based inserts had significantly medium uptake ability than CH/Carp based ones (*p* = 0.027). The related interaction between the carboxylate groups of carbopol with chitosan likely diminished hydrogen bond formation, which would lower the potential for hydration. This interaction masked the negative charge of the carboxylate moiety, resulting in difficulties in formation of secondary chemical bonds with water. These results are in accordance with SEM results, explaining superiority of CH/Alg and CH/Carp polymer complexation over CH/XG complexation [14]. However, no significant difference in medium uptake was observed between Na alginate and either xanthan gum or carbopol. Additionally, the effect of CH/anion ratio showed significant lowest medium uptake for 5:5 molar ratio of CH/anion in comparison with all other molar ratios at *p* < 0.05. This could be explained by minor amount of charges in CH/anion (5:5) resulting from maximized interaction between anion and chitosan, thus reducing medium uptake [16]. The opposite mechanism applies for the other molar ratios showing greater medium uptake due to greater amount of charges. Further comparisons showed that lower chitosan ratios (1:9 and 3:7) had greater medium uptake ability than those with higher chitosan ratio (9:1 and 7:3), respectively [16,18].

The measured detachment force for the prepared inserts ranged from 0.06 ± 0.01 to 0.23 ± 0.05 N for CH/Alg based vaginal inserts (F1–F5 formulae), 0.021 ± 1.004 to 0.080 ± 0.005 N for CH/XG based vaginal inserts (F6–F10 formulae) and 0.038 ± 1.003 to 0.098 ± 0.026 for CH/Carp based vaginal inserts (F11–F15 formulae). It was obvious that different quantities of chitosan significantly affected the mucoadhesion ability of the inserts.

### 3.5. In Vitro Release Studies and Kinetic Analysis

**Figure 4:**
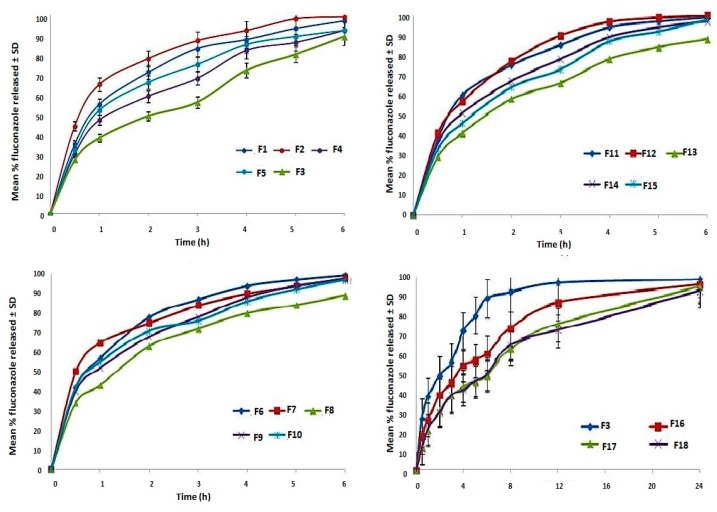
Fluconazole release from chitosan/anion polyelectrolyte based lyophilized vaginal inserts in phosphate buffer PH 4.5 (Data is represented as mean ± SD, n = 3).

The release behavior from fluconazole vaginal inserts in phosphate buffer pH 4.5 is illustrated in Figure 4. In vitro fluconazole release profiles of fluconazole-PECs based vaginal inserts were assessed by calculating the release efficiency after 6 h (RE_6h_) and time to T_d_. RE_6h_ ranged from 56.46 ± 3.42 to 79.38 ± 3.42% and Td ranged from 1.056 ± 1.03 to 3.011 ± 0.72 h Table 1.

To determine the release model of fluconazole, in-vitro release data were analyzed according to zero order, first order, Higuchi, Hixson Crowell and Weibull models [34,35]. The model showing the highest value of the coefficient of determination (R^2^) was considered the appropriate release model. Korsmeyer-Peppas model was excluded as it is valid only for the first 60% of release; whereas in our study most of vaginal insert exceeded this threshold in no more than three sampling points, which limited the applicability of the model [35]. Fluconazole drug release complied with Weibull model for all insert formulations, thus it was used to interpret the possible release mechanism of the drug. For all formulations, shape parameter (*β*) and time for release of 63.2% (T_d_) were calculated by KinetDS 3 rev 2010 software. As per literature, there is a linear relationship between the *β* and the n exponent used in Peppas model to determine release mechanism [34]. Several authors reported the interpretation of *β* value in terms of release mechanism as follows: Fickian diffusion (*β* ≤ 0.75), Fickian diffusion combined with case II transport (*β* 0.75–1.0) and complex release mechanism (*β* > 1.0) [35]. In the present study, *β* value was 0.75–1.0 for majority of inserts indicating Fickian diffusion combined with case II transport release mechanism; with the exception of F5, F8, F9, F13 where release mechanism was Fickian diffusion (*β* ≤ 0.75). The aforementioned results indicate gradual release of the drug from the prepared inserts with 60–80% total drug release after 4 h. The main mechanism of drug release was found to be Fickian diffusion.

### 3.6. Statistical Analysis of Factorial Design

Recognizing the variables that might influence the properties of drug delivery systems is crucial. Factorial designs are beneficial in this concern, as they are able to analyze the effect of various variables simultaneously. For the studied responses, results showed that predicted R^2^ values (0.7810, 0.7346 and 0.6296) agreed reasonably with the adjusted R^2^ (0.8910, 0.8679 and 0.8156) for RE_6h_%, Td h and mucoadhesion, respectively. Adequate precision with a ratio greater than four was detected in all responses, ensuring that suitability of the model to navigate the design space [22,36].

#### 3.6.1. Effect on Mucoadhesion

**Figure 5:**
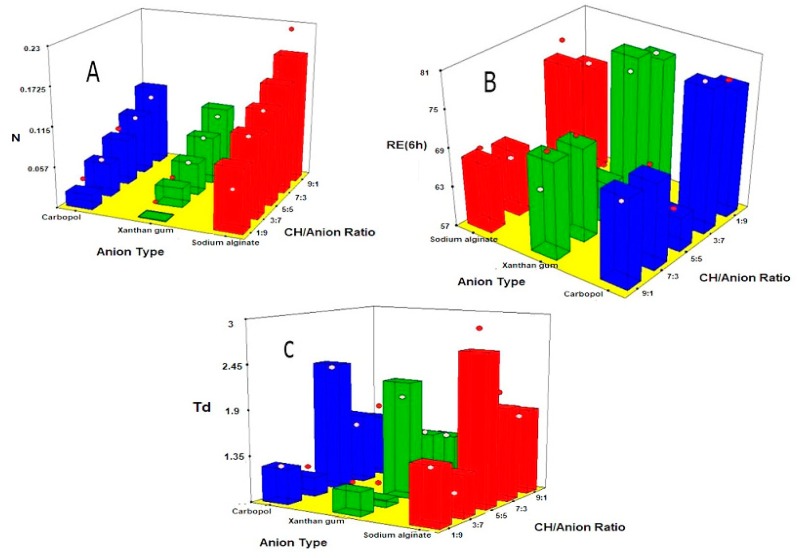
Response 3-D plots for effect of anion type (X_1_) and anion ratio (X_2_) on (**A**) mucoadhesion (**B**) release efficiency after 6 h (R_6h_); (**C**) time for 63.2% release (T_d_) from fluconazole chitosan/anion based vaginal inserts.

The influence of the anion type (X_1_), CH/anion ratio (X_2_) on the mucoadhesion of vaginal inserts is illustrated as 3-D surface plots in Figure 5A. ANOVA showed that both X1 (type of anion) and X_2_ (chitosan: anion ratio) had significant effect on mucoadhesion (*p* = 0.0101 and 0.0008, respectively).

Regarding the anion type, it was evident that CH/Alg based vaginal inserts showed the strongest mucoadhesion followed by carbopol, while xanthan showed the least mucoadhesion. According to previous studies, mucoadhesion effect increases with the number of hydrogen bonds (-OH, -COOH) involved in the mucoadhesion interaction. This explains superiority of Na Alginate (pK_a_ = 3.21) in comparison to carpobol (pK_a_ = 5) [16,20]. Paradoxically, xanthan gum, which has more hydrogen bonds (pK_a_ = 2.65) exhibited the weakest mucoadhesion profile. This could be probably explained by the helical three-dimensional structure of xanthan gum molecule preventing access to the charged mucin groups in the mucoadhesion interface [37]. Figure 5A showed that mucoadhesion increased proportionally with CH/anion ratio, with higher detachment force observed for greater chitosan content. This behavior might be interpreted by the existence of positively charged amino groups of chitosan at PH 4.5 that could interact with the negatively charged sialic acid (pK_a_ 2.6) and sulfate residues of the mucin glycoprotein [20].

#### 3.6.2. Effect on In Vitro Release

The effect of the anion type (X_1_), CH/anion ratio (X_2_) on the release parameters; RE_6h_ and T_d_ are graphically represented as 3-D surface plots in Figure 5B,C. ANOVA showed that both anion type (X_1_) and CH/anion ratio (X_2_) have significant effect on RE_6h_ (*p* = 0.0001 and 0.0327, respectively) and Td (*p*-value = 0.0003 and 0.0229, respectively). Polymers can be arranged in an escalating order according to their ability to sustain fluconazole release as xanthan gum < carbopol < Na alginate. This could be interpreted on the basis of interaction between cationic amino groups and anionic carboxylic groups. The superiority of Na alginate could be due to the lower pK_a_ of Alginate (3.21) compared to that of carbopol (5.0). However, xanthan gum exhibited the highest release despite of its lowest pK_a_ (2.62) owing to its weak interaction with chitosan. The previous results of DSC and SEM of PECs potentially support these results.

The effect of CH/anion ratio (X_2_) on drug release parameters was significant at *p* < 0.05. F3 (5:5 CH/Alg) showed the lowest RE_6h_ and longest T_d_. This could be due to strong interaction between chitosan cationic amino groups and alginate carboxylic groups with the subsequent inclusion of the drug into the formed PEC [16]. Furthermore, formulations with higher chitosan concentrations showed more sustained release profile compared to those with lower chitosan concentration. This might be attributed to the properties of chitosan including relatively high swelling rate and low medium uptake [18,38].

#### 3.6.3. Numerical Optimization

The results of numerical optimization revealed that the optimized formulation with maximized mucoadhesion, lowest RE_6h_ and highest T_d_ was F3 CH/Alg 5:5 with desirability of 0.87. The predicted values for RE_6h_% (59.2%), T_d_ (2.65 h) and mucoadhesion (0.139 N) were close to the determined values showing no statistically significant difference (*p* < 0.05), thus proving the reliability of the method.

### 3.7. Modulation of Release Profile

Different concentrations of Compritol 888 were added to impart hydrophobicity and retard drug release from the optimized vaginal insert F3 [14]. It was observed that adding Compritol reduced the initial amount released and retarded overall drug release rate [14,39]. The computed RE_6h_ for formulations F3, F16, F17 and F18 were 57.79 ± 0.94, 42.22 ± 0.13, 34.03 ± 0.52 and 33.86 ± 0.43, respectively.

Kinetic analysis of the release data of formulations F16, F17 and F18 were found to follow the Weibull model (showing highest R^2^ values of 0.9915, 0.9862, 0.9883, respectively). The computed *β* values for the three formulations lied between 0.7 and 1.0 indicating that the probable release mechanism is Fickian diffusion combined with case II transport. T_d_ for the aforementioned formulations were computed as 3.01, 5.36, 7.63 and 7.90 h, respectively. One-way ANOVA analysis revealed significant differences in RE_6h_ (*p* < 0.001) and Td (*p* < 0.001) among F3 and Compritol containing formulations. This finding confirmed the ability of Compritol to modulate drug release profile. Post hoc analysis showed significant differences in the release parameters between F16 and F17; whereas no significant difference was found between F17 and F18. Accordingly, F17 containing 1% Compritol was further characterized showing satisfactory drug content of 98 ± 0.013%, swelling index of 215.98 ± 2.12%, friability of 0.91 ± 1.49%, and mucoadhesion detachment force of 0.13 ± 0.01 N. The selected aforementioned formulation was subjected to further microbiological studies.

### 3.8. In Vitro Microbiological Assays

The antifungal activity of the selected fluconazole vaginal insert (F17) was assessed by comparing its inhibition zone against *C. albicans* with that of fluconazole solution and unloaded insert with the same composition as F17. Fluconazole vaginal insert showed the highest antifungal activity (mean inhibition zone of 31 ± 0.4 mm). This confirms the ability of the prepared inserts to enhance the antifungal activity of the drug compared to fluconazole solution (mean inhibition zone of 22 ± 0.4 mm) and drug-free insert (mean inhibition zone of 6 ± 0.5 mm). It is worthy to note that chitosan has reported fungistatic, explaining the presence of inhibition zone in drug-free inserts [40].

### 3.9. In-Vivo Microbiological and Histological Evaluation

The group in which fluconazole vaginal inserts were administered showed complete cure after 7 days for all the rats used. Among rats treated with fluconazole solution, only one rat had negative culture after 2 days of treatment and two rats after seven days. For the untreated group, all vaginal swabs were positive for *C. albicans* up to the twenty-first day. Negative culture was shown in only one rat from the unloaded vaginal insert group after the seventh day of treatment. Accordingly, complete microbiological cure was observed only with fluconazole PEC based vaginal insert (F17) as demonstrated in Table 2. Statistical analysis using One-way ANOVA revealed significant differences among groups at 95% confidence of level. Multiple comparisons according to Tukey’s test showed significantly lower microbial count (colony forming units) for the drug loaded PEC based inserts group in comparison to other groups (*p* > 0.05). This confirms the superiority of the fluconazole PECs based vaginal inserts in treating vaginal candidiasis and their ability to enhance the local action of the drug compared to fluconazole solution [24].

**Figure 6:**
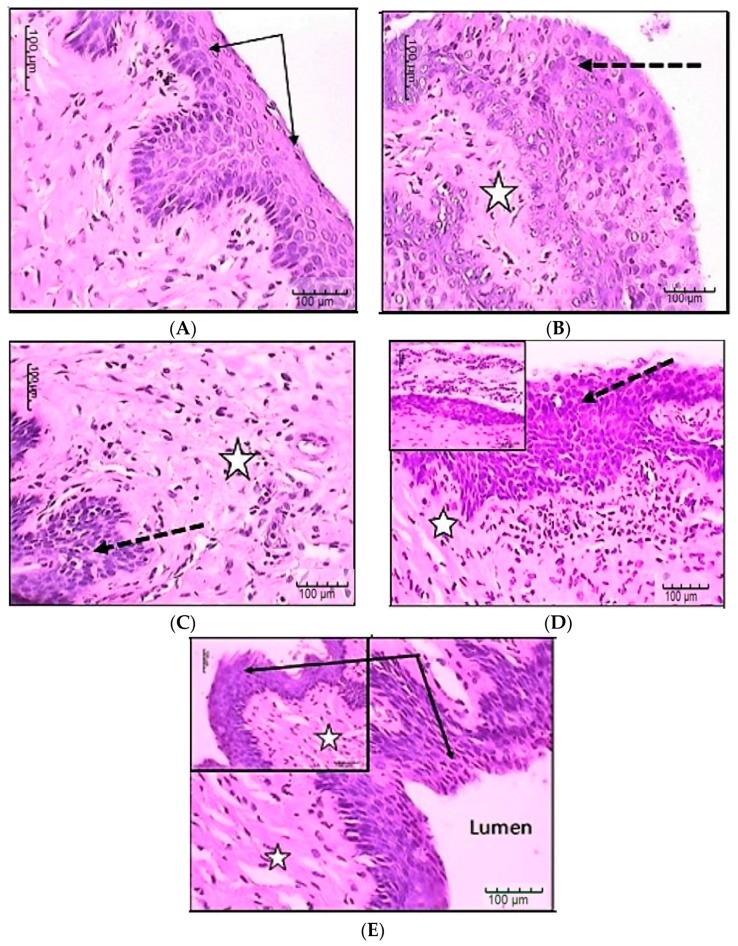
Histological examination of Candida infected vaginal tissue treated by unloaded vaginal insert, fluconazole PEC based vaginal insert and fluconazole solution. (**A**) Control normal vaginal tissue; (**B**) Control *Candida* infected, non-treated vaginal tissue; (**C**) *Candida* infected vaginal tissue treated by unloaded vaginal insert; (**D**) *Candida* infected vaginal tissue treated by fluconazole solution; (**E**) *Candida* infected vaginal tissue treated by fluconazole vaginal insert. Stars represent inflammatory cells; Black arrows represent normal epithelium; Dotted arrows represent hyperplastic or damaged epithelium.

Histopathological studies are shown in Figure 6 representing sections in female rat vagina stained by Hematoxylin and Eosin (H&E). Figure 6A presents normal control female rat vaginal tissue, showing intact normal stratified squamous, non-keratinized epithelial covering (black arrows) and underlying lamina propria with few connective tissue cells and fibers. Figure 6B presents control infected vaginal tissue of female rat showing hyperplastic epithelium with candida particles between cells and in the lamina, as well as mononuclear inflammatory cells in the lamina propria. Figure 6C presents vaginal tissue of female rat, which was treated by unloaded vaginal insert (F17); it shows focal areas of epithelial laceration with lamina propria with congested inflamed blood vessels as well as mononuclear inflammatory cells in the lamina propria. Figure 6D presents vaginal tissue of female rat treated by fluconazole solution; it shows hyperplastic epithelial covering or focal atrophy. Candida organisms mixed with inflammatory cells were also present on the lumen or lamina propria. Figure 6E presents vaginal tissue of female rat treated by selected fluconazole vaginal insert (F17); it shows nearly normal intact surface epithelium with normal thickness and only few inflammatory cells in lamina propria.

## 4. Conclusions

3^1^5^1^ factorial design was successfully applied for the formulation and optimization of fluconazole polyelectrolyte complex (PEC) based vaginal inserts. The optimized insert formulation containing chitosan/alginate (5:5) PEC exhibited controlled drug release, adequate mucoadhesion and consequently appropriate vaginal retention. In addition, it showed enhanced antifungal activity against Candida albicans both in vitro and in vivo with reduced inflammatory cells. Thus, the proposed PEC based vaginal insert could be a promising platform for vaginal delivery of antifungal drugs aiming at vaginal candidiasis treatment.

## Figures and Tables

**Table 1:** Composition and characterization of fluconazole polyelectrolyte complex based vaginal inserts prepared according 3^1^5^1^ full factorial design.

Run	Independent Variables		Responses (Dependent Variable)			Drug Content * (%)	Friability ^#^ (%)	Swelling Index * (%)
	Anion Type X_1_	Chitosan/Anion Ratio X_2_	Maximum Detachment Force ^$^ (N)	RE_6h_^$^ (%)	T_d_^$^ (h)			
F1	Alg	9:1	0.06 ± 0.010	73.62 ± 2.32	1.452 ± 0.65	102 ± 0.008	3.87 ± 1.46	191.24 ± 1.07
F2	Alg	7:3	0.11 ± 0.020	79.21 ± 3.22	1.056 ± 0.89	101 ± 0.033	3.54 ± 1.22	208.81 ± 2.03
F3	Alg	5:5	0.13 ± 0.010	56.46 ± 3.42	3.011 ± 1.22	98 ± 0.013	1.91 ± 1.49	215.98 ± 2.12
F4	Alg	3:7	0.18 ± 0.030	65.38 ± 2.87	2.097 ± 1.34	91 ± 0.054	2.12 ± 2.81	201.83 ± 3.09
F5	Alg	9:1	0.23 ± 0.050	69.51 ± 3.42	1.739 ± 0.87	98 ± 0.026	2.32 ± 2.01	215.98 ± 2.23
F6	XG	9:1	0.021 ± 0.004	78.27 ± 3.54	1.153 ± 0.67	100 ± 0.023	6.34 ± 2.91	192.34 ± 2.42
F7	XG	7:3	0.037 ± 0.004	77.63 ± 2.83	1.052 ± 0.86	93 ± 0.029	6.41 ± 4.96	209.61 ± 1.29
F8	XG	5:5	0.042 ± 0.025	65.25 ± 3.26	2.024 ± 1.52	94 ± 0.011	2.12 ± 1.49	209.61 ± 1.34
F9	XG	3:7	0.062 ± 0.003	71.60 ± 1.79	1.51 ± 1.13	95 ± 0.029	4.56 ± 2.51	199.92 ± 1.28
F10	XG	9:1	0.080 ± 0.005	72.65 ± 2.98	1.391 ± 0.92	98 ± 0.040	4.28 ± 1.39	204.87 ± 1.73
F11	Carp	9:1	0.038 ± 0.003	77.42 ± 2.37	1.224 ± 1.07	100 ± 0.023	3.23 ± 2.06	211.24 ± 1.39
F12	Carp	7:3	0.048 ± 0.007	79.38 ± 3.42	1.129 ± 1.11	102 ± 0.057	3.12 ± 2.83	201.83 ± 1.78
F13	Carp	5:5	0.079 ± 0.004	62.54 ± 1.97	2.321 ± 1.72	101 ± 0.013	2.16 ± 1.91	221.75 ± 2.05
F14	Carp	3:7	0.080 ± 0.004	72.13 ± 3.71	1.505 ± 0.74	97 ± 0.062	2.45 ± 1.87	211.25 ± 1.49
F15	Carp	9:1	0.098 ± 0.026	69.33 ± 2.36	1.687 ± 1.02	102 ± 0.018	2.34 ± 2.45	202.47 ± 2.91

Formula Composition (% *w*/*w*), CH (Chitosan), Alg (Na Alginate), XG (xanthan gum), Carp (carbopol), RE_6h_: Release efficiency after 6 h; T_d_: time after which drug release reached 63.2%. Data are presented as mean ± SD, ^$^ n = 3, * n = 5, ^#^ n = 2 (Test repeated twice, each time on 20 inserts).

**Table 2:** Viability of Candida albicans in swabs from vaginal rats inoculated with fluconazole loaded PECs based vaginal inserts (F17) in comparison to unloaded insert and fluconazole solution.

Time (day)	Control	Unloaded Insert	Fluconazole Loaded PEC Based Insert	Fluconazole Solution
T0	3.19 ± 3.03	2.8 ± 2.7	3.1 ± 2.9	2.79 ± 2.49
T2	2.97 ± 2.71	2.49 ± 2.35	3.08 ± 2.8	2.74 ± 2.58
T5	3.03 ± 3.17	2.19 ± 2.32	2.38 ± 2.2	2.99 ± 3.012
T7	2.5 ± 2.12	2.71 ± 2.85	-	2.93 ± 2.75
T21	1.87 ± 1.7	2.21 ± 2.83	-	2.62 ± 2.81

Results represent microbial concentration (log CFU ± SD) (n = 6). PEC: Polyelectrolyte complex; control: untreated group.
